# AMH in Males: Effects of Body Size and Composition on Serum AMH Levels

**DOI:** 10.3390/jcm12134478

**Published:** 2023-07-04

**Authors:** Veronika Tandl, Christoph Haudum, Katharina Eberhard, Barbara Hutz, Ines Foessl, Ewald Kolesnik, Andreas Zirlik, Dirk von Lewinski, Daniel Scherr, Nicolas Verheyen, Thomas Pieber, Barbara Obermayer-Pietsch

**Affiliations:** 1 Division of Endocrinology and Diabetology, Department of Internal Medicine, Medical University of Graz, 8036 Graz, Austria; veronika.tandl@medunigraz.at (V.T.);; 2 Core Facility Computational Bioanalytics, Medical University Graz, 8036 Graz, Austria; 3 Division of Cardiology, Department of Internal Medicine and University Heart Center Graz, Medical University of Graz, 8036 Graz, Austria

**Keywords:** anti-Müllerian hormone, hemodilution, body mass index, body composition

## Abstract

Serum concentrations of anti-Müllerian hormone (AMH) have been found to decrease with increasing body mass index (BMI) in many studies. It is not yet clear whether this stems from an adverse effect of adiposity on AMH production, or from dilution due to the greater blood volume that accompanies a larger body size. To investigate a possible hemodilution effect, we explored the relationships between serum AMH levels and different parameters of body composition using linear regression models in a cohort of adult males. Body weight, lean mass (LM), and body surface area (BSA) were found to be better predictors of AMH than measures of adiposity, such as BMI or fat mass. Since both LM and BSA correlate with plasma volume better than adipose tissue, we conclude that hemodilution of AMH does occur in adult males and should be considered for normalization in future studies.

## 1. Introduction

Anti-Müllerian hormone is a gonadal protein hormone, named after its role during male fetal development, when it leads to regression of the Müllerian ducts. In males, it is produced in the Sertoli cells in the seminiferous tubules of the testes [1,2]. In females, production takes place in the granulosa cells of growing follicles in the ovaries [3,4]. Although AMH is also expressed by some other organs, their contribution to circulating hormone levels is not noteworthy [5]. In clinical practice, AMH is used to differentiate between disorders of sex development and to assess endocrine Sertoli cell function, and it serves as a marker for functional ovarian reserve [2,3]. Even though AMH levels in males decrease after puberty, they are still detectable in considerable amounts (higher than in females) at older ages [1,6]. This indicates additional physiological roles that are yet unclear.

Anti-Müllerian hormone is well known in the context of polycystic ovary syndrome (PCOS). In PCOS, the serum levels of AMH are generally elevated, and AMH has even been implicated in the disorder’s development. Many of the morbidities associated with PCOS have also been found in first-degree male relatives of females with PCOS. Therefore, a male equivalent of PCOS is being discussed. There is some evidence indicating that AMH is overexpressed in affected males [5].

In several studies, body mass index (BMI) has been found to inversely correlate with serum AMH levels in males [7,8,9,10,11,12] and females, both with [13,14] and without PCOS [13,14,15,16,17]. Other studies have reported no such—or unclear—findings [18,19,20,21,22], leaving the relationship between BMI and serum AMH levels controversial. Some authors have argued that adiposity might adversely affect AMH-producing cells [7,9,10,16]. Among the mechanisms through which obesity might affect the hypothalamic–pituitary–gonadal axis (and thereby AMH production) are inflammatory mediators, hyperinsulinemia, oxidative stress, hyperthermia, and adipokines [23].

An alternative possibility is the dilution of AMH in the greater volume of plasma that accompanies a larger body size (known as hemodilution). Such a dilution effect has been found to be the reason underlying the negative relationship between different tumor markers and BMI [24,25,26]. 

with regard to AMH, it remains unclear whether the inverse relationship with BMI is because of a negative effect of obesity on AMH production, or if it stems from dilution in a greater blood volume. When researching AMH in serum, it is important to know whether the plasma volume is a confounder in order to avoid potential bias. Jaswa et al. [14] investigated a possible hemodilution effect of AMH in women with and without PCOS [14]. To the best of our knowledge, no such investigation has yet been conducted in males. 

The aim of this study was to explore the relationship between serum AMH levels and body composition in males and to investigate a possible hemodilution effect. For this, we used linear regression models of serum AMH levels and body composition parameters in a cohort of adult men. Body mass, lean mass (LM), and body surface area (BSA) were found to be better predictors of AMH than measures of adiposity. Since BSA and LM correlate with blood volume more strongly than adipose tissue [27], we conclude that the often observed inverse relationship between AMH and BMI is at least in part due to hemodilution. To avoid bias, such a dilution effect should be considered in future studies by normalizing to blood volume or a suitable proxy thereof. 

## 2. Materials and Methods

We used data from the BioPersMed cohort (Biomarkers of Personalized Medicine), a single-center, prospective, observational study at the Medical University of Graz (Graz, Austria). The participants were community-dwelling adults with at least one traditional cardiovascular risk factor (i.e., smoking, elevated total cholesterol, or arterial hypertension) but no manifestation of cardiovascular disease. They were recruited between 2010 and 2016, mainly via general practitioners, peripheral hospitals, and outpatient clinics at the Medical University of Graz. A cohort profile with details on recruitment, participants, and collected data has been published previously [28].

Blood samples for hormone measurements were taken after an overnight fast. Serum AMH levels were measured by immunoassay (Beckmann Coulter, Krefeld, Germany).The levels of luteinizing hormone (LH) and (FSH) were determined using Access hLH and hFSH CLIA (Beckman Coulter, Brea, CA, USA), while estradiol (E2) was measured with an IMMULITE CLIA assay (Siemens Healthcare Diagnostics Products, Munich, Germany) and free testosterone (FT) levels using an active Free Testosterone automated competitive immunoassay (Immunodiagnostic Systems, Frankfurt, Germany). Sex hormone-binding globulin (SHBG) was assessed using Elecsys ECLIA (Roche Diagnostics, Mannheim, Germany).

**Figure 1:**
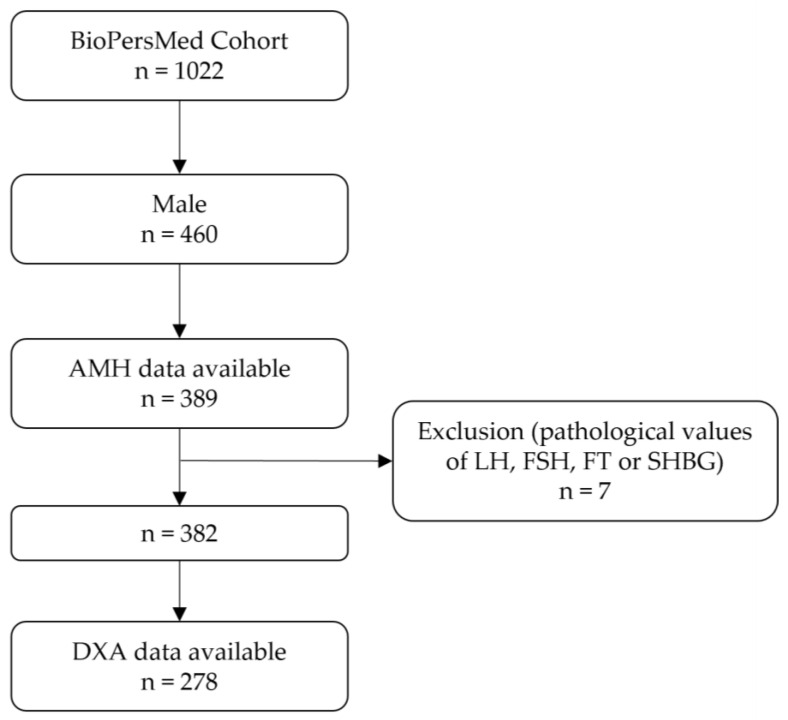
Flow chart of the study cohort. Abbreviations: AMH, anti-Müllerian hormone; LH, luteinizing hormone; FSH, follicle-stimulating hormone; FT, free testosterone; SHBG, sex hormone-binding globulin; DXA, dual-energy X-ray absorptiometry.

Of the 1022 enrolled study participants, 460 were males, of which 389 had serum AMH data available. Due to pathologically high values of LH, FSH, FT, or SHBG, seven participants were excluded, resulting in a study cohort of 382. Of these, 278 had DXA-derived body composition data available. A flow chart is presented in Figure 1. 

All participants had anthropometric data assessed and were asked about smoking and drinking habits as part of more extensive questionnaires. Height and weight, as well as hip and waist circumference (WC) measurements, were taken by trained personnel. Body composition was determined using dual-energy X-ray absorptiometry (DXA) with Lunar iDXA (General Electrics, Madison, WA, USA). Among the DXA-derived parameters were fat mass (FM), lean mass (LM), android fat mass (AFM), and gynoid fat mass (GFM). The latter two describe fat mass at the waist (AFM) and hip (GFM) regions. Body mass index (BMI) was calculated as weight (kg) divided by height squared (m^2^). Waist–hip ratio (WHR) refers to waist and hip circumference. Android–gynoid ratio (AGR) is AFM divided by GFM. High WHR and AGR indicate central adiposity. Fat mass index (FMI) and lean mass index (LMI) were calculated similarly to BMI, but using FM and LM instead of weight, respectively. We used the Du Bois equation [29] to estimate body surface area (BSA) using weight and height. For estimating the body fat percentage, we chose the Clínica Universidad de Navarra-Body Adiposity Estimator (CUN-BAE), which correlates with cardiometabolic risk even better than BMI [30]. To calculate the estimated LM (eLM), we used the equation of Janmahasatian et al. [31]. In a comparison of different equations for estimating LM, this one has been found to be optimal for adults [32]. The measured and calculated body composition parameters are summarized in Table 1.

The descriptive study sample characteristics are given as medians (interquartile ranges (IQRs)). Normality was tested using the Kolmogorov–Smirnov test. Groups were compared with the Mann–Whitney *U*-test. Linear regression models were used to assess the relationship between serum AMH levels and body composition parameters, with *p* < 0.05 considered statistically significant. Assumptions for linear regression were tested beforehand. All models had normal distribution of residuals and a variance inflation factor (VIF) < 2.5, indicating no collinearity. We performed univariate regressions (model 1), as well as multivariate models with potential confounders included as additional predictors. Model 2 included age, while model 3 included age, FSH, and estradiol (E2). These confounders were chosen since in preliminary analyses, serum FSH and E2 were found to be the strongest predictors of AMH in the study cohort, whereas smoking and alcohol were not significantly associated with serum AMH levels (Table S1). AMH levels did not follow a normal distribution, and instead were positively skewed. To achieve normal distribution, the values were log-transformed after adding a constant of 0.93. The transformed values were used for all of the regression models. In the regression coefficients, interpretation of the quantitative changes and units of the response variable were not straightforward, as the AMH values were not back-transformed. Thus, interpretation was limited to the direction (positive or negative) and significance of the relationships. All analyses were performed using R Statistical Software (v4.2.2) [33].

## 3. Results

### 3.1. Study Characteristics

Descriptive statistics for the study cohort are summarized in Table 2. To make sure that missingness of DXA data was random and did not introduce bias, the subset of participants with available DXA measurements (“DXA subset”, *n* = 278) was contrasted to the whole study cohort (*n* = 382). There were no statistically significant differences between the two. The median age of the whole cohort was 59 years (IQR: 13). The AMH values ranged from 0.07 to 23.00 ng/mL, with a median of 4.75 ng/mL (IQR: 4.10). 

### 3.2. Relationships between Serum AMH Levels and DXA-Derived Body Composition Parameters

To investigate the relationships between AMH levels and body composition, we first used the DXA-derived parameters in the “DXA subset” (*n* = 278) of the cohort (Table 3). Different linear regression models were employed. Model 1 was a univariate model, whereas the others were multivariate, including the potential confounders age (model 2) or age, FSH, and E2 (model 3). 

Scatterplots, visualizing the relationships between DXA-derived body composition and serum AMH, are shown in Figure S1. Models 1 and 2 showed similar results. The parameters indicative of adiposity (FM; model 1: *R*^2^ = 0.013; β = −0.003; *p* = 0.0313; model 2: *R*^2^ = 0.035; β = −0.003; *p* = 0.0234) and central adiposity (AFM; model 1: *R*^2^ = 0.013; β = −0.023; *p* = 0.0343; model 2: *R*^2^ = 0.032; β = −0.023; *p* = 0.0372), as well as those indicative of lean mass (LM; model 1: *R*^2^ = 0.022; β = −0.005; *p* = 0.0074; model 2: *R*^2^ = 0.063; β = −0.008; *p* = 0.0003 and LMI; model 1: *R*^2^ = 0.011; β = −0.017; *p* = 0.0438; model 2: *R*^2^ = 0.039; β = −0.021; *p* = 0.0125), each had significant negative relationships with serum AMH levels. However, in model 3 (including the potential confounders age, FSH, and E2), the results for FM, AFM, and LMI were no longer significant. Only for LM did the statistical significance persist (*R*^2^ = 0.197; β = −0.006; *p* = 0.003). No significant relationships were found with FMI and AGR in any model. 

To further investigate whether LM (as a proxy of blood volume) or FM better predicts serum AMH levels, they were both included in model 2, thus being challenged against each other. Of the two predictors, only LM remained significant (*R*^2^ = 0.061; β = −0.007; *p* = 0.0035), while FM did not (Table 4). These results indicate that LM is a better predictor of serum AMH levels than measures of adiposity.

### 3.3. Relationships between Serum AMH Levels and General Body Composition Parameters

Body composition analysis by DXA requires the appropriate appliance and trained personnel. Consequently, this means that DXA-derived data were not available in every study. We therefore investigated if the results of the DXA-derived body composition parameters could be reproduced with anthropometric measures that are more commonly employed. For these analyses, the whole study cohort (*n* = 382) was used. 

The results are summarized in Table 5. The scatterplots corresponding to model 1 are shown in Figure S2. There was no statistically significant relationship between WHR and serum AMH levels. In both models 1 and 2, measures of overall mass (weight; model 1: *R*^2^ = 0.034; β = −0.003; *p* = 0.0002; model 2: *R*^2^ = 0.062; β = −0.004; *p* < 0.0001 and BSA; model 1: *R*^2^ = 0.035; β = −0.027; *p* = 0.0001; model 2: *R*^2^ = 0.068; β = −0.319; *p* < 0.0001), adiposity (BMI; model 1: *R*^2^ = 0.022; β = −0.009; *p* = 0.0021; model 2: *R*^2^ = 0.043; β = −0.009; *p* = 0.0021, WC; model 1: *R*^2^ = 0.028; β = −0.004; *p* = 0.0007; model 2: *R*^2^ = 0.04; β = −0.003; *p* = 0.0025 and CUN-BAE; model 1: *R*^2^ = 0.028; β = −0.008; *p* = 0.0007; model 2: *R*^2^ = 0.039; β = −0.007; *p* = 0.0044), and lean mass (eLM; model 1: *R*^2^ = 0.034; β = −0.007; *p* = 0.0002; model 2: *R*^2^ = 0.067; β = −0.008; *p* < 0.0001) had significant negative relationships with AMH. Mirroring the results of the “DXA subset” analysis, parameters indicative of adiposity did not retain significance in model 3 (accounting for age, FSH, and E2). Only weight (*R*^2^ = 0.201; β = −0.002; *p* = 0.0022), BSA (*R*^2^ = 0.206; β = −0.231; *p* = 0.0006), and eLM (*R*^2^ = 0.206; β = −0.006; *p* = 0.0006) remained significant.

Using model 2 (adjusted for age), both eLM and BSA were challenged against BMI or CUN-BAE (Table 6). Similar to LM in the “DXA subset,” the parameters eLM and BSA were used as estimators of blood volume, while BMI and CUN-BAE were used as measures of adiposity. Neither BMI nor CUN-BAE remained statistically significant in the models that included eLM or BSA. The estimated LM (model including BMI: *R*^2^ = 0.065; β = −0.008; *p* = 0.0018; model including CUN-BAE: *R*^2^ = 0.066; β = −0.009; *p* = 0.0008) and BSA (model including BMI: *R*^2^ = 0.065; β = −0.331; *p* = 0.0013; model including CUN-BAE: *R*^2^ = 0.066; β = −0.354; *p* = 0.0006), however, did retain significance.

## 4. Discussion

We investigated the serum AMH levels and body composition in adult males. Several measures of body size had a negative relationship with AMH. Body mass, LM, and BSA were better predictors of AMH than measures of adiposity, such as BMI or fat mass. These results indicate that AMH concentrations are lower in the greater blood volume resulting from a larger body size, especially increased LM. 

Different measures of adiposity were used in our study. Total FM, BMI, FMI, and CUN-BAE were used as measures of overall adiposity. All but FMI had a negative relationship with serum AMH levels, which is in accordance with numerous other publications [7,8,9,10,11,12,13,14,15,16,17]. However, these results were significant only in the univariate and age-adjusted models (models 1 and 2, respectively). They did not remain significant in the fully adjusted model (model 3), which accounted for age, FSH, and E2, suggesting them as confounders. 

Android FM, AGR, WC, and WHR describe central adiposity. Accumulation of adipose tissue around the abdomen is particularly associated with health complications as a result of obesity [34]. In our cohort, AGR and WHR could not significantly predict AMH in any of the regression models used. Android FM and WC were significant in models 1 and 2 only, similar to the other adiposity parameters. Therefore, our data do not indicate a negative effect on AMH production by adiposity per se.

As described by Boer [27] in 1984, blood volume can be estimated by body weight, BSA, or, preferably, LM. Adipose tissue, which is only poorly perfused, adds relatively little to plasma volume. In our cohort, weight, BSA, LM, and eLM were significantly inversely related to serum AMH. They were the only parameters that remained significant in all regression models used, regardless of potential confounders.

In addition, when we challenged the estimators of blood volume (BSA, LM, and eLM) against measures of adiposity (FM, BMI, and CUN-BAE) in age-adjusted regression models (model 2), only the former remained significant, while the latter did not. These results support the hemodilution hypothesis. 

Together, these results support the notion that there was a dilution effect of AMH in the greater blood volume in our cohort of adult males. However, this does not preclude a negative effect of adiposity on AMH production by Sertoli cells altogether. Rather, normalizing AMH concentrations to blood volume (or a proxy thereof) might strengthen research investigating serum AMH levels in both males and females, whether researching possible systemic, physiological functions of the hormone or in context of PCOS and the male PCOS equivalent. 

Using DXA-derived body composition data is probably ideal, but in many studies, only simple anthropometrics such as height, weight, and sometimes WC are collected. Thus, we used not only DXA data, but more available body composition parameters for our analyses, yielding comparable results. In our study, there were similar results for both eLM and BSA. Since BSA can introduce errors in extreme sizes such as morbid obesity, the use of eLM is preferable [35]. This might be particularly important when studying serum AMH levels in PCOS, which is often accompanied by obesity [36].

Jaswa et al. [14] conducted a study on AMH, BMI, and a possible hemodilution in women with and without PCOS [14]. In both groups, they found that BMI, but not BSA, was associated with decreased serum AMH concentrations, supporting a non-dilutional reduction of AMH. Although the authors provided several possible explanations of how adiposity might affect AMH production by granulosa cells, these concepts cannot be easily transferred to males because of sexual dimorphism. In addition, matters regarding AMH are complicated by ovarian cycle and follicle number and stage in females, especially with PCOS [37]. 

Of note, Pietiläinen et al. [8] stated that in their cohort of adult men, negative correlations with AMH were significant for BMI and fat mass, but not lean mass. However, adjustments for age, FSH, and E2 were not included and the sample size, 64 twin pairs, was smaller than in our cohort, which might contribute to the different findings. Moreover, investigating hemodilution per se was not the aim of their research, but rather investigating the genetic and environmental components of AMH levels in healthy men in general. 

The main strength of our study is the use of DXA measurements for body composition in addition to conventional anthropometric data. In this study, we used regression models that are either univariate or include age or FSH, age, and E2. These models allowed a deeper insight into the relationship between AMH and body composition. However, there might still be other confounders affecting serum AMH concentrations—a limitation of this study. Furthermore, the study cohort comprised only adult men with cardiovascular risk factors (but no manifestation of cardiovascular disease), with other populations left to be considered. In addition, blood volume was not directly quantified, but body mass and composition parameters as a proxy were used instead. Another limitation of this study is its retrospective, cross-sectional design. 

There are several caveats to consider regarding AMH dilution in blood volume. Body size and composition are not the only factors influencing AMH levels. Age, health status, genetics, and other hormones have effects on AMH production [1,2,6,8,38]. Additionally, body size and lean mass are not the only contributors to plasma volume [24].

## 5. Conclusions

We found that in adult males, body mass, body surface area, and estimated or measured lean mass better predicted serum AMH levels than measures of adiposity. This suggests that dilution in a greater blood volume is at least in part responsible for the often-observed inverse relationship between serum AMH concentrations and BMI. Hemodilution should therefore be considered for normalization in future studies by using estimated or measured lean mass as a proxy for blood volume.

## Figures and Tables

**Table 1:** Body composition parameters.

Parameter	Measurement/Calculation
Height	Directly measured (m)
Weight	Directly measured (kg)
Hip circumference	Directly measured (cm)
Waist circumference (WC)	Directly measured (cm)
Fat mass (FM)	DXA (kg)
Lean mass (LM)	DXA (kg)
Android fat mass (AFM)	DXA (kg)
Gynoid fat mass (GFM)	DXA (kg)
Body mass index (BMI)	weight (kg)/height^2^ (m^2^)
Waist–hip ratio (WHR)	waist (cm)/hip (cm)
Android–gynoid ratio (AGR)	AFM (kg)/GFM (kg)
Fat mass index (FMI)	FM (kg)/height^2^ (m^2^)
Lean mass index (LMI)	LM (kg)/height^2^ (m^2^)
Body surface area (BSA)	Du Bois equation [29]
Body Adiposity Estimator (CUN-BAE)	CUN-BAE equation [30]
Estimated lean mass (eLM)	Janmahasatian equation [31]

Abbreviations: AFM, android fat mass; GFM, gynoid fat mass; FM, fat mass; LM, lean mass; CUN-BAE, Clínica Universidad de Navarra-Body Adiposity Estimator.

**Table 2:** Study cohort characteristics.

	Whole Cohort (*n* = 382)	“DXA Subset” (*n* = 278)	*p*
AMH (ng/mL)	4.75 (4.10)	4.87 (4.61)	0.504
Age (years)	59 (13)	59 (13)	0.838
Smoking (*n*)	48 (13%)	35 (13%)	
Alcohol (dpw)	3 (6)	3 (6)	0.929
FSH (µU/mL)	7.17 (5.16)	7.07 (5.31)	0.749
E2 (pg/mL)	34.4 (18.5)	34.7 (18.7)	0.822
Weight (kg)	84.95 (17.80)	84.45 (17.07)	0.742
Height (cm)	177.00 (8.20)	176.80 (8.67)	0.486
WC (cm)	94.00 (14.75)	93.00 (15.00)	0.576
WHR	0.97 (0.07)	0.97 (0.07)	0.985
BMI	26.87 (5.19)	26.87 (5.10)	0.981
Fat mass (kg)	-	23.87 (11.33)	-
Lean mass (kg)	-	57.39 (8.93)	-

Variables are given as the median (IQR) or *n* (%). A Mann–Whitney *U*-test was used to compare the whole cohort and the subset for which DXA-derived data were available (“DXA subset”). Abbreviations: AMH, anti-Müllerian hormone; dpw, drinks per week; FSH, follicle-stimulating hormone; E2, estradiol; WC, waist circumference; WHR, waist–hip ratio; BMI, body mass index; IQR, interquartile range.

**Table 3:** Relationships between AMH levels and DXA-derived body composition parameters.

	Model 1			Model 2			Model 3		
	Univariate			Multivariate Age			Multivariate Age, FSH, E2		
	*R* ^2^	β	*p*	*R* ^2^	β	*p*	*R* ^2^	β	*p*
FM	0.013	−0.003	**0.0313**	0.035	−0.003	**0.0234**	0.177	−0.002	0.1407
FMI	0.009	−0.009	0.0613	0.029	−0.009	0.0664	0.173	−0.004	0.3503
AFM	0.013	−0.023	**0.0343**	0.032	−0.023	**0.0372**	0.175	−0.013	0.2051
AGR	−0.003	−0.023	0.734	0.017	−0.009	0.8901	0.173	0.053	0.3904
LM	0.022	−0.005	**0.0074**	0.063	−0.008	**0.0003**	0.197	−0.006	**0.003**
LMI	0.011	−0.017	**0.0438**	0.039	−0.021	**0.0125**	0.178	−0.012	0.1079

Goodness-of-fit (*R*^2^), linear regression coefficient (β), and *p*-value are given for the univariate models (model 1), as well as the multivariate models that include the potential confounders age (model 2) or age, FSH, and E2 (model 3) as additional predictors. Significant *p*-values in bold. Transformed AMH values were used (see methods). “DXA subset” (*n* = 278). Abbreviations: AGR, android–gynoid ratio; FMI, fat mass index; LMI, lean mass index.

**Table 4:** Relationship between FM or LM and AMH in a multivariate linear regression model.

	Model 2 Multivariate Age		
	*R* ^2^	β	*p*
	0.061		
FM		−0.001	0.4988
LM		−0.007	**0.0035**

Goodness-of-fit (*R*^2^), linear regression coefficient (β), and *p*-value are given for a multivariate model that includes the potential confounder age (model 2) and challenges LM and FM against each other. Transformed AMH values were used (see methods). “DXA subset” (*n* = 278). Significant *p*-values in bold.

**Table 5:** Relationships between AMH levels and general body composition parameters.

	Model 1			Model 2			Model 3		
	Univariate			Multivariate Age			Multivariate Age, FSH, E2		
	*R* ^2^	β	*p*	*R* ^2^	β	*p*	*R* ^2^	β	*p*
Weight	0.034	−0.003	**0.0002**	0.062	−0.004	**<0.0001**	0.201	−0.002	**0.0022**
BSA	0.035	−0.027	**0.0001**	0.068	−0.319	**<0.0001**	0.206	−0.231	**0.0006**
BMI	0.022	−0.009	**0.0021**	0.043	−0.009	**0.0021**	0.189	−0.005	0.0581
WC	0.028	−0.004	**0.0007**	0.04	−0.003	**0.0025**	0.189	−0.002	0.0815
WHR	0.002	−0.264	0.188	0.018	−0.2	0.3179	0.184	−0.129	0.4816
CUN-BAE	0.028	−0.008	**0.0007**	0.039	−0.007	**0.0044**	0.187	−0.004	0.0837
eLM	0.034	−0.007	**0.0002**	0.067	−0.008	**<0.0001**	0.206	−0.006	**0.0006**

Goodness-of-fit (*R*^2^), linear regression coefficient (β), and *p*-value are given for univariate models (model 1), as well as multivariate models that include the potential confounders age (model 2) or age, FSH, and E2 (model 3) as additional predictors. Transformed AMH values were used (see methods). Significant *p*-values in bold. Abbreviations: BSA, body surface area; CUN-BAE, Clínica Universidad de Navarra-Body Adiposity Estimator; eLM, estimated lean mass. Whole study cohort (*n* = 382).

**Table 6:** Multivariate linear regression models challenging either eLM or BSA against BMI or CUN-BAE.

	Model 2 Multivariate Age				Model 2 Multivariate Age		
	*R* ^2^	β	*p*		*R* ^2^	β	*p*
	0.065				0.066		
BMI		5 × 10^−5^	0.9885	BMI		0.001	0.8693
eLM		−0.008	**0.0018**	BSA		−0.331	**0.0013**
	0.065				0.066		
CUN-BAE		0.001	0.7382	CUN−BAE		0.002	0.6306
eLM		−0.009	**0.0008**	BSA		−0.354	**0.0006**

Goodness-of-fit (*R*^2^), linear regression coefficient (β), and *p*-value are given for a multivariate model that includes the potential confounder age (model 2) and challenges LM and FM against each other. Significant *p*-values in bold. Transformed AMH values were used (see methods). Whole study cohort (*n* = 382).

## Data Availability

Currently no public data available due to contract reasons.

## Supplementary Materials

The following supporting information can be downloaded at: https://www.mdpi.com/article/10.3390/jcm12134478/s1, Figure S1: Scatterplots of DXA-derived parameters and serum AMH levels with regression line; Figure S2: Scatterplots of commonly available body composition parameters and serum AMH levels with regression line; Table S1: Relationships of potential confounders with serum AMH levels—preliminary analysis.

## References

[1] Edelsztein N.Y., Valeri C., Lovaisa M.M., Schteingart H.F., Rey R.A. (2022). AMH Regulation by Steroids in the Mammalian Testis: Underlying Mechanisms and Clinical Implications. Front. Endocrinol..

[2] Grinspon R., Rey R. (2010). Anti-Müllerian Hormone and Sertoli Cell Function in Paediatric Male Hypogonadism. Horm. Res. Paediatr..

[3] Moolhuijsen L.M.E., Visser J.A. (2020). Anti-Müllerian Hormone and Ovarian Reserve: Update on Assessing Ovarian Function. J. Clin. Endocrinol. Metab..

[4] di Clemente N., Racine C., Pierre A., Taieb J. (2021). Anti-Müllerian Hormone in Female Reproduction. Endocr. Rev..

[5] di Clemente N., Racine C., Rey R.A. (2022). Anti-Müllerian Hormone and Polycystic Ovary Syndrome in Women and Its Male Equivalent. Biomedicines.

[6] Aksglaede L., Sørensen K., Boas M., Mouritsen A., Hagen C.P., Jensen R.B., Petersen J.H., Linneberg A., Andersson A.-M., Main K.M. (2010). Changes in Anti-Müllerian Hormone (AMH) throughout the Life Span: A Population-Based Study of 1027 Healthy Males from Birth (Cord Blood) to the Age of 69 Years. J. Clin. Endocrinol. Metab..

[7] Robeva R., Tomova A., Kirilov G., Kumanov P. (2011). Anti-Müllerian Hormone and Inhibin B Levels Reflect Altered Sertoli Cell Function in Men with Metabolic Syndrome. Andrologia.

[8] Pietiläinen K.H., Kaprio J., Vaaralahti K., Rissanen A., Raivio T. (2012). Circulating Anti-Müllerian Hormone Levels in Adult Men Are under a Strong Genetic Influence. J. Clin. Endocrinol. Metab..

[9] Andersen J.M., Herning H., Aschim E.L., Hjelmesæth J., Mala T., Hanevik H.I., Bungum M., Haugen T.B., Witczak O. (2015). Body Mass Index Is Associated with Impaired Semen Characteristics and Reduced Levels of Anti-Müllerian Hormone across a Wide Weight Range. PLoS ONE.

[10] Buyukinan M., Atar M., Pirgon O., Kurku H., Erdem S.S., Deniz I. (2018). Anti-Mullerian Hormone and Inhibin B Levels in Obese Boys; Relations with Cardiovascular Risk Factors. Exp. Clin. Endocrinol. Diabetes.

[11] Beydoun H.A., Hossain S., Beydoun M.A., Weiss J., Zonderman A.B., Eid S.M. (2019). Anti-Müllerian Hormone Levels and Cardiometabolic Disturbances by Weight Status Among Men in the 1999 to 2004 National Health and Nutrition Examination Survey. J. Endocr. Soc..

[12] Hadlow N.C., Brown S.J., Lim E.M., Prentice D., Pettigrew S., Cronin S.L., Prescott S.L., Silva D., Yeap B.B. (2022). Anti-Müllerian Hormone Concentration Is Associated with Central Adiposity and Reproductive Hormones in Expectant Fathers. Clin. Endocrinol..

[13] Moslehi N., Shab-Bidar S., Ramezani Tehrani F., Mirmiran P., Azizi F. (2018). Is Ovarian Reserve Associated with Body Mass Index and Obesity in Reproductive Aged Women? A Meta-Analysis. Menopause.

[14] Jaswa E.G., Rios J.S., Cedars M.I., Santoro N.F., Pavone M.E.G., Legro R.S., Huddleston H.G. (2020). Increased Body Mass Index Is Associated with A Nondilutional Reduction in Antimüllerian Hormone. J. Clin. Endocrinol. Metab..

[15] Bernardi L.A., Carnethon M.R., de Chavez P.J., Ikhena D.E., Neff L.M., Baird D.D., Marsh E.E. (2017). Relationship Between Obesity and Anti-Müllerian Hormone in Reproductive-Aged African-American Women. Obesity.

[16] Ou M., Xu P., Lin H., Ma K., Liu M. (2021). AMH Is a Good Predictor of Metabolic Risk in Women with PCOS: A Cross-Sectional Study. Int. J. Endocrinol..

[17] Buyukkaba M., Turgut S., Ilhan M.M., Ekinci I., Yaylım İ., Zeybek S.U., Turan S., Tasan E., Karaman O. (2022). Anti-Mullerian Hormone Levels Increase After Bariatric Surgery in Obese Female Patients with and without Polycystic Ovary Syndrome. Horm. Metab. Res..

[18] Tüttelmann F., Dykstra N., Themmen A.P.N., Visser J.A., Nieschlag E., Simoni M. (2009). Anti-Müllerian Hormone in Men with Normal and Reduced Sperm Concentration and Men with Maldescended Testes. Fertil. Steril..

[19] Sahmay S., Usta T., Erel C.T., İmamoğlu M., Küçük M., Atakul N., Seyisoğlu H. (2012). Is There Any Correlation between Amh and Obesity in Premenopausal Women?. Arch. Gynecol. Obstet..

[20] Moy V., Jindal S., Lieman H., Buyuk E. (2015). Obesity Adversely Affects Serum Anti-Müllerian Hormone (AMH) Levels in Caucasian Women—PMC. J. Assist. Reprod. Genet..

[21] Oldfield A.L., Kazemi M., Lujan M.E. (2021). Impact of Obesity on Anti-Mullerian Hormone (AMH) Levels in Women of Reproductive Age. J. Clin. Med..

[22] Rerat S., Amsellem-Jager J., L’hour M.C., Bouhours-Nouet N., Donzeau A., Rouleau S., Levaillant L., Emeriau F., Moal V., Boux de Casson F. (2022). Lower Circulating Sertoli and Leydig Cell Hormone Levels During Puberty in Obese Boys: A Cross-Sectional Study. J. Clin. Endocrinol. Metab..

[23] Bellastella G., Menafra D., Puliani G., Colao A., Savastano S. (2019). How Much Does Obesity Affect the Male Reproductive Function?. Int. J. Obes. Suppl..

[24] Fowke J.H., Matthews C.E. (2010). PSA and Body Composition by Dual X-ray Absorptiometry (DXA) in NHANES. Prostate.

[25] Song M., Doo S.W., Yang W.J., Song Y.S., Kim Y. (2010). Serum Prostate-Specific Antigen Is Better Correlated to Body Surface Area than Body Mass Index in a Population of Healthy Korean Men. Int. J. Urol..

[26] Park M., Chang H.I., Kang H., Han S.S. (2015). Effect of Obesity-Related Plasma Hemodilution on Serum Tumor Marker Concentration in Women. J. Obstet. Gynaecol. Res..

[27] Boer P. (1984). Estimated Lean Body Mass as an Index for Normalization of Body Fluid Volumes in Humans. Am. J. Physiol.-Ren. Physiol..

[28] Haudum C.W., Kolesnik E., Colantonio C., Mursic I., Url-Michitsch M., Tomaschitz A., Glantschnig T., Hutz B., Lind A., Schweighofer N. (2022). Cohort Profile: Cohort Profile: ‘Biomarkers of Personalised Medicine’ (BioPersMed): A Single-Centre Prospective Observational Cohort Study in Graz/Austria to Evaluate Novel Biomarkers in Cardiovascular and Metabolic Diseases. BMJ Open.

[29] Du Bois D., Du Bois E. (1989). A Formula to Estimate the Approximate Surface Area If Height and Weight Be Known. 1916. Nutrition.

[30] Gómez-Ambrosi J., Silva C., Catalán V., Rodríguez A., Galofré J.C., Escalada J., Valentí V., Rotellar F., Romero S., Ramírez B. (2012). Clinical Usefulness of a New Equation for Estimating Body Fat. Diabetes Care.

[31] Janmahasatian S., Duffull S.B., Ash S., Ward L.C., Byrne N.M., Green B. (2005). Quantification of Lean Bodyweight. Clin. Pharmacokinet..

[32] Puri T., Blake G.M. (2022). Comparison of Ten Predictive Equations for Estimating Lean Body Mass with Dual-Energy X-ray Absorptiometry in Older Patients. Br. J. Radiol..

[33] R Core Team (2021). R: A Language and Environment for Statistical Computing.

[34] Goossens G.H. (2017). The Metabolic Phenotype in Obesity: Fat Mass, Body Fat Distribution, and Adipose Tissue Function. Obes. Facts.

[35] Peters A.M., Snelling H.L.R., Glass D.M., Love S., Bird N.J. (2010). Estimated Lean Body Mass Is More Appropriate than Body Surface Area for Scaling Glomerular Filtration Rate and Extracellular Fluid Volume. Nephron Clin. Pract..

[36] Barber T.M., Franks S. (2021). Obesity and Polycystic Ovary Syndrome. Clin. Endocrinol..

[37] McLennan I.S., Pankhurst M.W. (2017). Is the Understanding of AMH Being Confounded by Study Designs That Do Not Adequately Reflect That It Is an Atypical Hormone?. Hum. Reprod..

[38] Eckersten D., Giwercman A., Bruun L., Christensson A. (2015). Anti-Müllerian Hormone, a Sertoli Cell-Derived Marker, Is Decreased in Plasma of Male Patients in All Stages of Chronic Kidney Disease. Andrology.

