# Supplementary material for: AMH in Males: Effects of Body Size and Composition on Serum AMH Levels
Source: J Clin Med. 2023 Jul 4;12(13):4478. doi: 10.3390/jcm12134478 (PMC10342968; doi:10.3390/jcm12134478)
Supplement: Supplementary file 1 [file jcm-12-04478-s001.zip › jcm-2424267-supplementary.pdf]

Table S1. Relationships of potential confounders with serum AMH levels – preliminary analysis

	Univariate linear regression model		
	**R^2**	**β**	***p***
age	.021	-.004	**<.0001**
FSH	.157	-.017	**<.0001**
E2	.051	-.003	**<.0001**
Alcohol [dpw]	-.001	-.0001	.448
Non-smoking	-.004	-.001	.731

* Goodness-of-fit (R^2), linear regression coefficient (β) and *p*-value are given for a univariate linear regression model. Transformed AMH values were used (see methods). Abbreviations: FSH, follicle-stimulating hormone; E2, estradiol; dpw, drinks per week. Study cohort (n = 382).

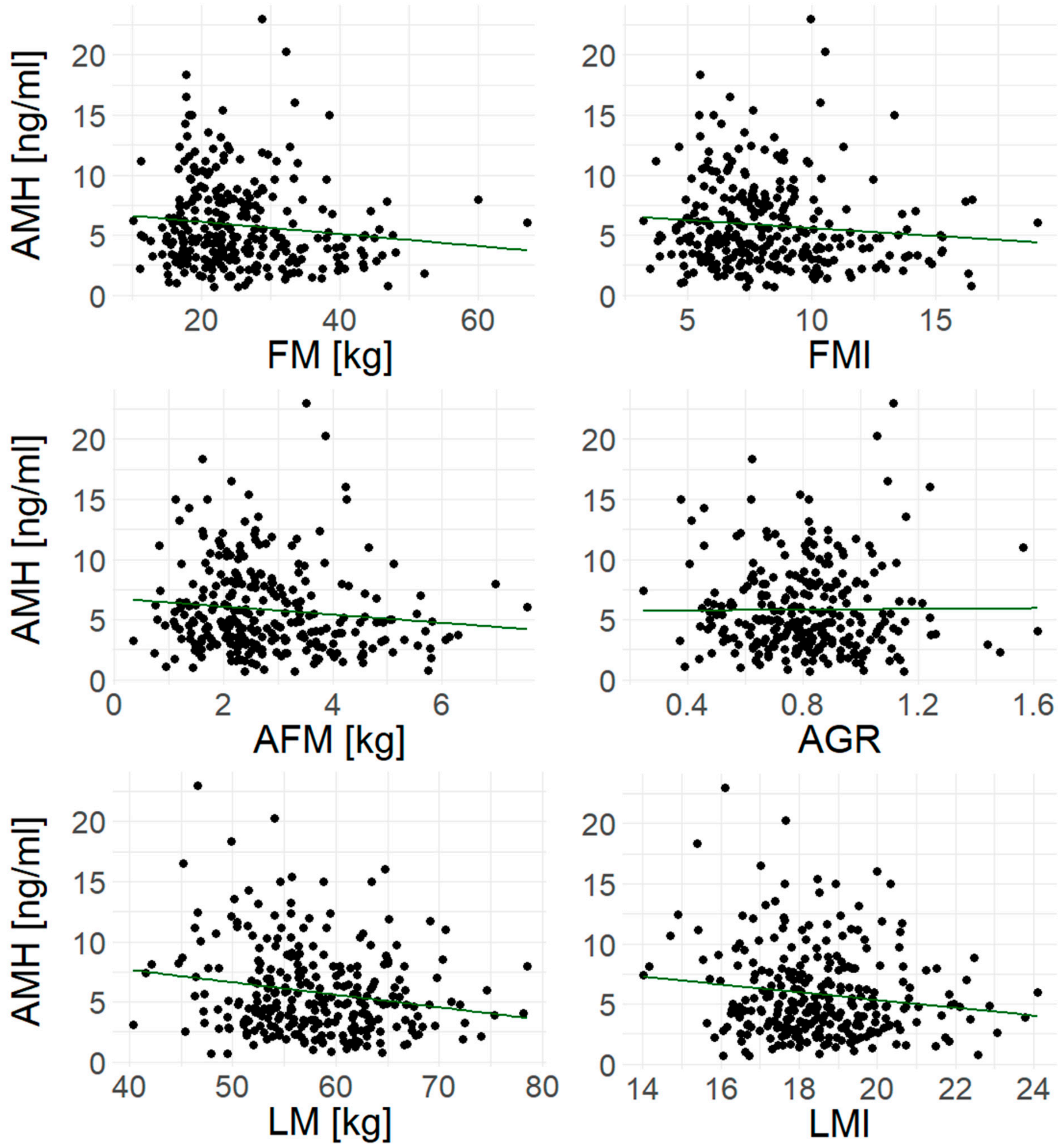

Figure S1. Scatterplots of DXA-derived parameters and serum AMH levels with regression line (green). "DXA subset" (n = 278). Abbreviations: FM, fat mass; FMI, fat mass index; AFM, android fat mass; AGR, android-gynoid ratio; LM, lean mass; LMI, lean mass index.

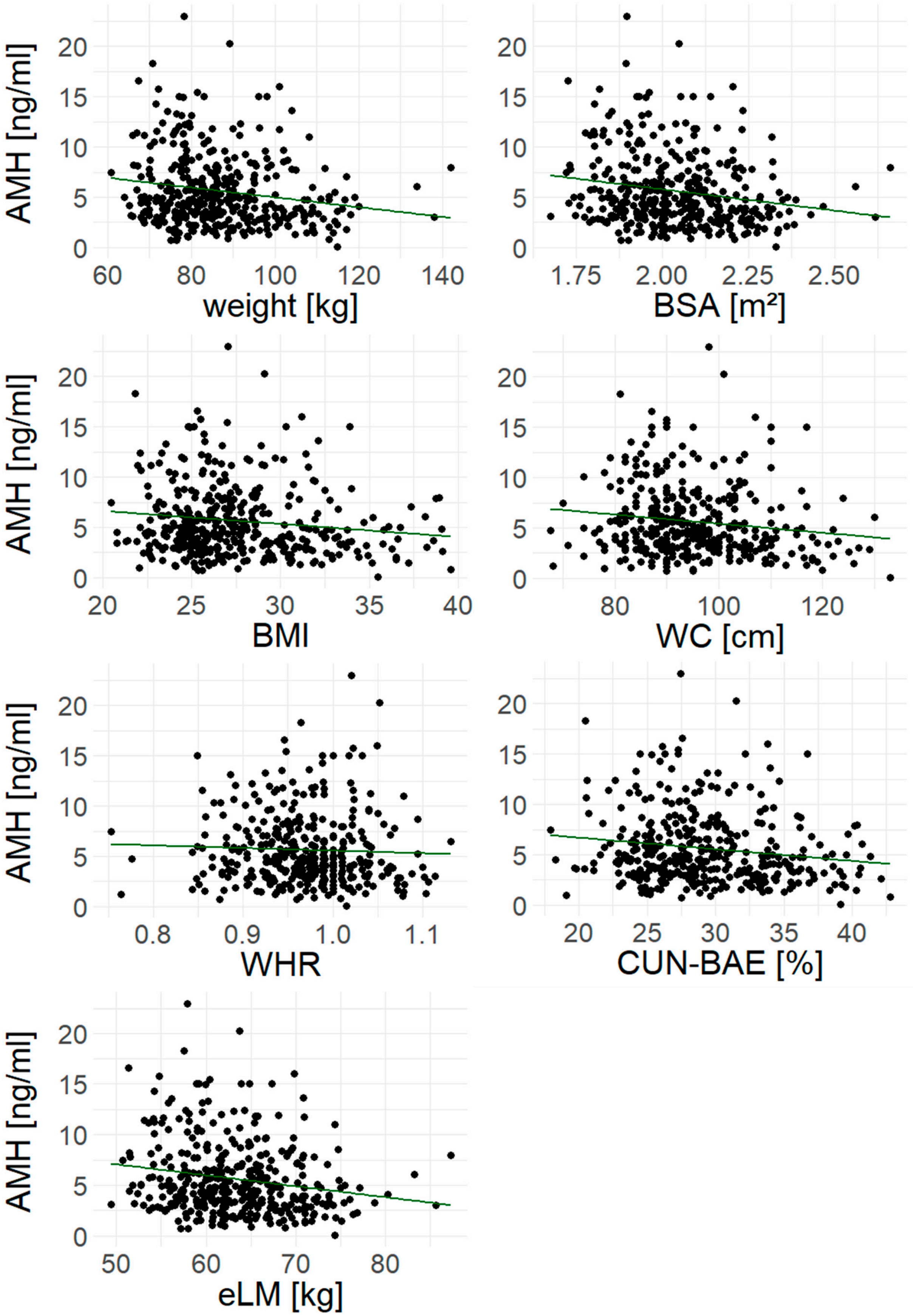

Figure S2. Scatterplots of commonly available body composition parameters and serum AMH levels with regression line (green). Whole cohort (n = 382). Abbreviations: BSA, body surface area; BMI, body mass index; WC, waist circumference; WHR, waist-hip ratio; CUN-BAE, Clínica Universidad de Navarra- Body Adiposity Estimator; eLM, estimated lean mass.
